# Recombinant heat shock protein 70 functional peptide and alpha-fetoprotein epitope peptide vaccine elicits specific anti-tumor immunity

**DOI:** 10.18632/oncotarget.12464

**Published:** 2016-10-04

**Authors:** Xiao-Ping Wang, Qiao-Xia Wang, Huan-Ping Lin, Bing Xu, Qian Zhao, Kun Chen

**Affiliations:** ^1^ Laboratory of Molecular Biology & Pathology, Shaanxi University of Chinese Medicine, Xianyang, PR China; ^2^ Department of Infectious Disease, Xi'an Central Hospital, School of Medicine, Xi'an Jiaotong University, Xi'an, PR China

**Keywords:** alpha-fetoprotein, antigen epitope, heat shock protein 70, functional peptide, immunity

## Abstract

Alpha-fetoprotein (AFP) is a marker of hepatocellular carcinoma (HCC) and serves as a target for immunotherapy. However, current treatments targeting AFP are not reproducible and do not provide complete protection against cancer. This issue may be solved by developing novel therapeutic vaccines with enhanced immunogenicity that could effectively target AFP-expressing tumors. In this study, we report construction of a therapeutic peptide vaccine by linking heat shock protein 70 (HSP70) functional peptide to the AFP epitope to obtain HSP70-P/AFP-P. This novel peptide was administered into BALB/c mice to observe the effects. Quantification of AFP-specific CD8 ^+^ T cells that secrete IFN-γ in these mice via ELISPOT revealed the synergistic effects of HSP70-P/AFP-P with increased numbers of AFP-specific CD8 ^+^ T cells. Similarly, ELISA analysis showed increased granzyme B and perforin released by natural killer cells. Moreover, *in vitro* cytotoxic T-lymphocyte assays and *in vivo* tumor preventive experiments clearly showed the higher antitumor effects of HSP70-P/AFP-P against AFP-expressing tumors. These results show that treatment of BALB/c mice with HSP70-P/AFP-P induced stronger T-cells responses and improved protective immunity. Our data suggest that HSP70-P/AFP-P may be used as a therapeutic approach in the treatment of AFP-expressing cancers.

## INTRODUCTION

Cancer is one of the leading causes of deaths worldwide. Among the various cancer types, hepatocellular carcinoma (HCC) accounts for about 1.2 million deaths annually [1]. This malignancy is becoming more prevalent in Asia because of the high incidences of hepatitis B and C infections [1,2]. Current treatments for HCC largely involve surgery and liver transplantation but most patients lose their battle against HCC due to late diagnosis and development of cirrhosis [3]. Therefore, there is an urgent need to develop effective therapies that may prolong the life and improve the quality of patients with HCC.

Recently, there has been an increase in the development of therapeutic vaccines, which are expected to boost the host's immune system and target specific tumor-antigens. Thus, therapeutic vaccines can be considered for the treatment of HCC because about 80% of HCC patients express high levels of the alpha-fetoprotein (AFP), which can serve as a target for such immunotherapy [3,4]. AFP is an oncofetal protein that is expressed robustly during the development of HCC. However, current treatments targeting AFP are generally weak and do not provide reliable antitumor protection [5]. A number of therapeutic approaches have been described to improve pre-existing antitumor immunity. These include recombinant plasmid DNA, chimeric virus-like particles, viral or bacterial vectors expressing AFP proteins and adoptive transfer of tumor-specific T cells [4–11]. However, therapeutic vaccination has been limited due to the low response of the immune system to AFP antigen. Therefore, there is a need to boost the host's immune response against AFP by improving the immunogenicity of AFP.

Heat shock proteins (HSPs) are well known to play the role of chaperones during various cellular functions such as protein folding, degradation and transport. Moreover, they are also efficient in antigen presentation to CD8 ^+^ T cells through the MHC-I pathway [12]. Because of their dual function, HSPs can potentially serve as immunoadjuvants to enhance antigen-specific tumor immunity [12–14]. HSP-based tumor vaccines have been effective in treating tumors in animal models and are being testing in clinical trials [15,16]. Therefore, HSP can serve as an adjuvant to enhance the antitumor effects of immunotherapy targeting AFP-expressing tumors.

In this study, we constructed a peptide vaccine named HSP70-P/AFP-P by linking the AFP peptide (FMNKFIYEI) to the HSP70 functional peptide (TKDNNLLGRFELSG) and tested the hypothesis that the immunogenicity of AFP can be improved by HSP70-P/AFP-P due to the synergistic actions of the peptides. Administration of HSP70-P/AFP-P into BALB/c mice revealed significant increases in AFP-specific CD8 ^+^ T cell numbers, and improved responses of natural killer cells and antitumor effects against AFP-expressing tumors. These results suggest that the novel vaccine created by conjugating the HSP70 functional peptide with AFP epitope peptide may effectively prime mice by inducing a robust protective immunity against AFP, thus effectively killing AFP-expressing tumors.

## RESULTS

### Characterization of purified HSP70-P/AFP-P vaccine

**Figure 1:**
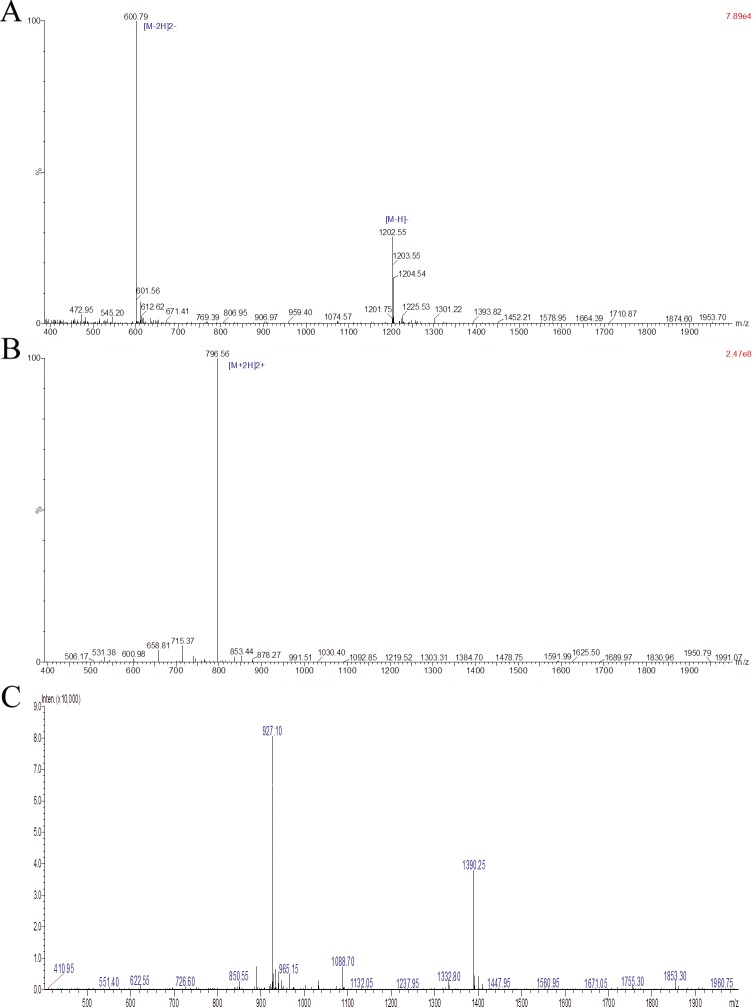
Analysis of purified HSP70-P/AFP-P by LC-MS The identity of the purified peptide was verified by LC-MS. **A**, Purified AFP peptide FMNKFIYEI. **B**, Purified HSP70 peptide TKDNNLLGRFELSG. **C**, Purified conjugated HSP70 peptide and AFP peptide TKDNNLLGRFELSGFMNKFIYEI.

The synthesized HSP70-P, AFP-P and HSP70-P/AFP-P vaccines were purified, analyzed and characterized by RP-HPLC chromatogram and LC-MS (Table 1, Figure 1). The results verified the successful construction and purification of the HSP70-P/AFP-P vaccine.

**Table 1:** Parameters of purified HSP70-P, AFP-P and HSP70-P/AFP-P

Groups	AFP-P	HSP70-P	HSP70-P/AFP-P
Molecular Weight	1204.46	1591.76	2778.21
MS Analysis	1202.55(M-H)^−^ 600.79(M-2H) ^2−^	796.56(M+2H) ^2+^	1390.25(M+2H) ^2+^ 927.10(M+3H) ^3+^
Peptide Purity	98.27%	98.54%	98.17%

### Priming mice with HSP70-P/AFP-P increased immune responses *in vivo*

To investigate the protective effects of HSP70-P/AFP-P, we first administered the AFP peptide vaccine into BALB/c mice. Then, 2 weeks after the last dose we measured the numbers of AFP-specific CD8 ^+^ T cells by using the ELISPOT assay. Stimulation of isolated CD8 ^+^ T cells with the AFP peptide showed a significant 26-fold increase in the numbers of AFP-specific IFN-γ-secreting CD8 ^+^ T cells (478 cells/10^6^ CD8 ^+^ T cells) only in the HSP70-P/AFP-P administered mice in comparison with the three remaining control groups that received PBS, AFP-P or HSP-P (17-18 cells/10^6^ CD8 ^+^ T cells/group) (*P* < 0.01) (Table 2). These results suggested that HSP70-P/AFP-P vaccine elicited a strong CTL response.

To determine whether natural killer cells participated in specific anti-tumor response, we measured the concentrations of granzyme B and perforin released from the natural killer cells harvested from mice immunized with HSP70-P/AFP-P and the controls. The results revealed that granzyme B and perforin concentrations produced by the natural killer cells from mice that received HSP70-P/AFP-P immunization were 12-fold higher in comparison with the control mice (Table 2) (*P* < 0.01). These results indicated that HSP70-P/AFP-P vaccine significantly induced the response of AFP-specific natural killer cells.

### HSP70-P/AFP-P immunization enhanced AFP-specific antibody production *in vivo*

Quantification of anti-AFP antibodies was determined by ELISA. Two weeks after vaccination revealed significantly higher levels of anti-AFP antibodies (10-fold) in the HSP70-P/AFP-P vaccinated group when compared to the control groups that received PBS, AFP-P or HSP70-P (*P* < 0.01) (Table 2). This result indicated that conjugating the AFP peptide with the HSP70 functional peptide increased the immunogenicity of AFP as evidenced by the increased levels of anti-AFP antibodies when compared to vaccinating the AFP peptide alone. However, the levels of anti-HSP70 antibody in all four mice groups remained the same indicating that the HSP70 peptide adjuvant was not immunogenic by itself (*P* > 0.05) (Table 2).

**Table 2:** Immunological parameters of mice immunized with therapeutic peptide vaccines

Groups	PBS	AFP-P	HSP70-P	HSP70-P/AFP-P
Spots^a^ (× 10^6^ cells)	17.35±7.42	18.32±7.55^#^	17.74±8.27^$^	478.65±16.75^‡^*
Granzyme^b^ (pg/ml)	8.47±2.26	8.37±2.34^#^	8.49±2.63^$^	104.73±8.62^‡^*
Perforin^c^ (pg/ml)	7.78±2.32	8.25±2.45^#^	8.38±2.54^$^	97.78±7.74^‡^*
AFP Abs^d^ (μg/ml)	1.40±0.38	1.41±0.26^#^	1.43±0.25^$^	14.76±3.22^‡^*
HSP70 Abs^e^ (μg/ml)	1.41±0.22	1.42±0.32^#^	1.46±0.34^$^	1.48±0.37^‡^*

a AFP-specific IFN-γ-secreting CD8^+^ T cells;

b Granzyme B released from natural killer cells;

c Perforin released from natural killer cells;

d AFP-P specific antibodies in mice;

e HSP70-specific antibodies in mice

# *P*>0.05, vs PBS group;

$ P>0.05, vs PBS group;

‡ *P*<0.01, vs AFP-P group;

* *P*<0.01, vs HSP70-P group.

### HSP70-P/AFP-P increased AFP-specific CD8 ^+^ T cell and natural killer cell responses

**Figure 2:**
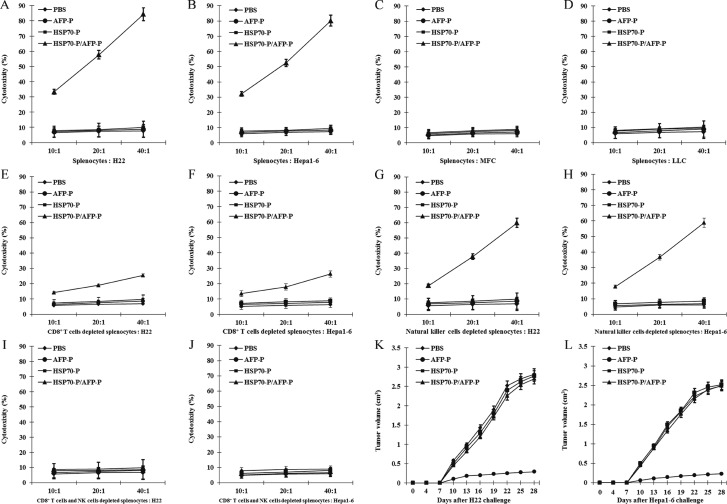
HSP70-P/AFP-P vaccine primed the strongest AFP-specific CD8**^+^** T cell and natural killer cell responses AFP-specific cytolytic activity was assayed against tumor cells at 10:1, 20:1 and 40:1 E/T (effector/target) ratios for H22 cells (**A**), Hepa1-6 cells (**B**), MFC cells (**C**) and LLC cells (**D**), CD8^+^ T cells depleted splenocytes for H22 cells and Hepa1-6 cells (**E**, F), natural killer cells depleted splenocytes for H22 cells and Hepa1-6 cells (**G**, **H**), both CD8^+^ T cells and natural killer cells depleted splenocytes for H22 cells and Hepa1-6 cells (**I**, **J**). Preventive immunization of mice with HSP70-P/AFP-P enhanced the anti-tumor immunity more significantly than immunization with AFP-P, HSP70-P or PBS (*P* < 0.01) (**K**, **L**).

Previous studies have shown that prophylactic or therapeutic treatment of mice tumors with HSP70/AFP vaccination can regress AFP-expressing tumors [10,17]. Therefore, in this study we investigated whether HSP70-P/AFP-P vaccination can induce similar effects by performing *in vitro* lymphocyte cytotoxicity assays. Isolated splenocytes from mice were stimulated *in vitro* with the AFP peptide followed by analysis of viable effector cells for cytotoxic activity against Hepa1-6 or H22 tumor cells. Significantly stronger cytotoxic effects on Hepa1-6 or H22 cells were observed in mice vaccinated with HSP70-P/AFP-P in comparison with the control mice that received PBS, AFP-P or HSP70-P (P < 0.01) (Figure 2A, 2B). More importantly, this cytotoxic effect was targeted toward the Hepa1-6 or H22 cells but not toward LLC or MFC cells (P < 0.01) (Figure 2C, 2D). These results clearly showed that the conjugation of AFP peptide with HSP70 peptide is a requirement to enhance specific CD8 ^+^ T cell and natural killer cell activity, since HSP70 or AFP peptides alone induced only low cytotoxic activity.

To distinguish whether natural killer cells or CD8 ^+^ T cells induced the cytotoxic effects on H22 or Hepa1-6 tumor cells, natural killer cell and CD8 ^+^ T cell depletion assays were performed. After depletion of natural killer cells or CD8 ^+^ T cells, reduced cytotoxic effects on Hepa1-6 or H22 cells were observed in the remaining splenocytes from mice vaccinated with HSP70-P/AFP-P, particularly after CD8 ^+^ T cells depletion. Cytotoxic effects were the lowest in the group where both natural killer cells and CD8 ^+^ T cells were depleted. In the control groups treated with PBS, AFP-P or HSP70-P cytotoxic effects of the splenocytes remained the same and did not differ significantly (*P >* 0.05) (Figure 2E-2J). These results indicated that vaccination with HSP70-P/AFP-P triggered both natural killer cell- and CD8 ^+^ T cell-induced cytotoxic activity against H22 or Hepa1-6 tumor cells.

### HSP70-P/AFP-P vaccination induced protective immunity against H22 or Hepa1-6 tumors

**Figure 3:**
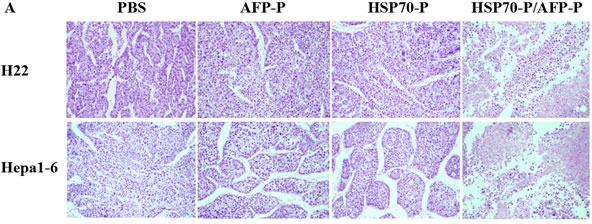

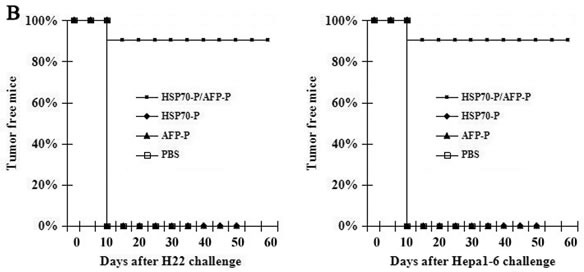
HSP70-P/AFP-P vaccination induced protective effect against H22 or Hepa1-6 tumors **A**, HSP70-P/AFP-P vaccination induced necrosis and lymphocyte infiltration of H22 or Hepa1-6 tumors, HE staining, × 200. **B**, Vaccination with HSP70-P/AFP-P eradicated pre-existing H22 or Hepa1-6 tumors. The mice immunized with HSP70-P or AFP-P died before day 50, and the mice immunized with HSP70-P/AFP-P still survived at day 60, whereas all the mice treated with PBS demised before day 35.

To further investigate the AFP-specific CTL response of HSP70-P/AFP-P vaccination, we examined the protective effect of the HSP70-P/AFP-P peptide vaccine in regressing pre-existing AFP-expressing Hepa1-6 or H22 tumors *in vivo*. First, female and male BALB/c mice were injected with 5 × 10^5^ Hepa1-6 or H22 tumor cells s.c. in the left flank followed by vaccination s.c. in the right flank with PBS, AFP-P, HSP70-P or HSP70-P/AFP-P (100 μl/mouse) on days 3, day 10 and day 17. The mice vaccinated with HSP70-P/AFP-P remained tumor-free for more than 60 days and only 2 mice developed a small tumor on day 10. In contrast, mice that received AFP-P or HSP70-P peptides showed significant tumor growth on day 10 after inoculation indicating that conjugation of AFP-P with HSP70-P is essential to reduce tumor growth. Mean tumor volumes in mice that received AFP-P or HSP70-P peptides alone were significantly higher than mice that were vaccinated with HSP70-P/AFP-P (*P* < 0.05) (Figure 2K, 2L). No statistically significant difference was observed in the tumor mass among the control groups that received HSP70-P, AFP-P or PBS. Similarly, necrosis and lymphocyte infiltration were higher in mice vaccinated with the HSP70-P/AFP-P peptide (Figure 3A) particularly in comparison with the group that received only the AFP-P peptide (*P* < 0.01) indicating the therapeutic effect of Hepa1-6 or H22 tumors by HSP70-P/AFP-P. Survival of mice also differed between the four treatment groups. Mice vaccinated with HSP70-P/AFP-P survived the longest for 60 days while mice that received HSP70-P or AFP-P peptides died before day 50 and all mice that received PBS died before day 35 (Figure 3B). Together, these data suggested that HSP70-P/AFP-P vaccination can exert a desirable protective effect against Hepa1-6 or H22 tumor cells *in vivo.* Vaccination with HSP70-P/AFP-P can not only reduce tumor size but also prolong the survival time of mice with tumors significantly better than vaccination with AFP-P alone.

## DISCUSSION

In general, AFP is produced only at low levels after birth, however, majority of human HCC overexpress AFP, which has a molecular weight of 64,000~72,000 kDa [3,4]. Recent studies have dissected the immunodominant epitopes of AFP in order to effectively use this protein for immunotherapy of AFP presenting tumors. Despite exposure to high plasma levels of AFP during hepatocellular carcinoma development, only low immunity is mounted against the protein [18,19]. A recent study identified four antigenic peptides in the human AFP that were recognized by the T cells *in vitro* to generate AFP-specific CTL [6]. Similar responses were also observed in murine models, which mounted a T cell response against AFP. Thus, AFP could be a promising candidate for use in immunotherapy. However, studies have reported the low immunogenicity and reproducibility of AFP-based vaccines that do not provide optimal protective immunity [4,5].

In this study, we developed a novel therapeutic vaccine to enhance the immunogenicity of AFP by conjugating AFP peptide with the HSP70 functional peptide via peptide synthesis. Our results showed that vaccination of mice with the novel HSP70-P/AFP-P vaccine mounted strong natural killer cell and CD8 ^+^ T cell responses that together provided a protective effect against AFP-producing tumors. These results also indicate that AFP-specific natural killer cell and CD8 ^+^ T cell responses contributed to the anti-tumor effects of HSP70-P/AFP-P vaccines. Besides, the HSP70-P/AFP-P vaccine also induced detectable levels of anti-AFP antibodies. Although the precise role of humoral immunity in combating tumors is not known, we presume that anti-AFP antibody may likely neutralize the AFP antigen or activate an antibody-dependent immune cell cytotoxicity.

HSP70 had been previously used as an adjuvant to enhance antigen-specific tumor immunity [20,21]. The functional peptide of HSP70, TKDNNLLGRFELSG (TKD), was reported to enhance the cytotoxic activity against HSP70-positive tumors via the NK cells [22–25]. In a previous study, we introduced HSP70 and several AFP peptides into a eukaryotic expression vector and then injected the recombinant vector into mice, which showed robust protective immunity [10]. Consistent with our findings others have also reported that conjugating HSP70 to target antigens can improve the potency of AFP fusion vaccines [18]. Therefore, synthesizing the HSP70-P/AFP-P peptide vaccine in this study is an innovative approach that holds great promise to induce antigen-specific T cell-mediated responses as well as humoral responses for cancer vaccine development.

Some studies have shown that since HSPs could induce cross-reactive T-lymphocytes, using HSPs for vaccination may increase the risk of autoimmunity [26–28]. However, we have shown in this study that using the functional peptide of HSP alone with the desired antigen such as AFP peptide did not cause life-threatening biological changes in organs such as heart, lung, live or kidney. More importantly, the absence of anti-HSP70 antibody in the HSP70-P/AFP-P and HSP70-P treated mice clearly shows the low immunogenicity of this HSP70 peptide thus explaining the lack of autoimmunity development.

Our data demonstrated that HSP70-P/AFP-P vaccination induced robust AFP-specific immune responses that resulted in the regression of established H22 or Hepa1-6 tumors. This could have resulted from a “cross-priming” mechanism [25,29,30] that may have enhanced the AFP-specific CD8 ^+^ T cells and natural killer cells to a very high level. Cross-priming occurs in different intracellular compartments where antigens are processed by enzymes [28]. It depends mostly on the characteristics of the antigen and the capacity of APCs [30]. Along these lines, HSPs have been shown to chaperone antigens into the MHC class-I APCs that may induce tumor-directed CD8 ^+^ CTL responses [31,32]. The “cross-priming” mechanism may explain the enhanced AFP-specific CD8 ^+^ T cell responses in mice vaccinated with HSP70-P/AFP-P. Moreover, HSPs function via various receptors to exert their effects [33–35]. Specifically, HSP70 has been shown to enhance NK cell cytotoxic responses by binding with the NKG2D receptor [25,36,37]. Our study revealed that HSP70-P/AFP-P vaccination elicited significant cytotoxicity of NK cells against H22 or Hepa1-6 tumor cells through granzyme B and perforin release.

In conclusion, our results indicate that HSP70-P/AFP-P vaccine can mount an impressive antitumor effect against AFP-expressing tumors in mice by enhancing the immune responses activated by AFP-specific CD8 ^+^ T cells and natural killer cells. Therefore, conjugating tumor antigen peptide with HSP70 functional peptide may be a promising immunotherapy for other cancers with known tumor-specific antigens.

## MATERIALS AND METHODS

### Mice and cell culture

Six- to eight-week-old male and female BALB/C mice were purchased from the Experimental Animal Center at Fourth Military Medical University. All animals were maintained under specific-pathogen-free conditions, and all procedures were performed according to approved protocols and in accordance with recommendations for the proper care of laboratory animals. The investigation was approved by the Ethics Committee on Animal Study at Shaanxi University of Chinese Medicine (2004-4B). H22 and Hepa1-6 mice hepatocellular carcinoma cells were kindly provided by School of Medicine, Xi'an Jiaotong University and maintained in RPMI 1640 (Gibco-BRL, USA) with 10% fetal bovine serum, 100 U/ml penicillin, and 100 μg/ml streptomycin (Invitrogen Corp., CA, USA) at 37°C under humidified atmosphere of 95% air and 5% CO_2_. Mouse gastric carcinoma cells (MFC) and Lewis lung carcinoma cells (LLC) were purchased from Institute of Biochemistry and Cell Biology of Chinese Academy of Sciences and cultured in DMEM (Gibco-BRL, USA) with 10% fetal bovine serum at 37°C in 5% CO_2_. The AFP concentration of 1 × 10^6^ H22 cultured cells was 241.5 ± 19.7 ng/ml, while the AFP concentration of 1 × 10^6^ Hepa1-6 cultured cells was 252.6 ± 21.4 ng/ml. AFP was not detected in MFC and LLC cells.

### AFP peptide, HSP70 peptide and conjugation

The strategy was designed to construct a peptide vaccine, combining HSP70 functional peptide, a C-terminal substrate-binding domain, with N-terminal AFP epitope peptide (−CO-NH-, amido linkage) by Fmoc solid phase peptide synthesis [38–40]. Mouse AFP peptide containing epitope 158-166 amino acid, FMNKFIYEI (AFP-P), HSP70 14-mer peptide TKDNNLLGRFELSG (HSP70-P) and recombinant peptide vaccine TKDNNLLGRFELSGFMNKFIYEI (HSP70-P/AFP-P) were synthesized from the ZiYuPeptides Co., Ltd (Shanghai, China). Lyophilized material was resuspended in sterile distilled water at 10 mg/ml, aliquoted, and stored at −70°C until use.

The identity of the purified peptide was verified by RP-HPLC chromatogram and LC-MS analysis (ABI, CA, USA). Protein concentration was determined using BCATM protein assay kit (Pierce Inc., Rockford, IL, USA).

### Mice immunized with HSP70-P/AFP-P vaccine

Male and Female BALB/C mice were randomly divided into HSP70-P/AFP-P group, AFP-P group, HSP70-P group and PBS control group. Every group had 20 mice. Before injection, each antigen was diluted in saline to the concentration of 100 μg/100μl. Each antigen was injected into the left flank of mice subcutaneously (s.c.). Priming and boosting was performed with 10μg HSP70-P/AFP-P, AFP-P or HSP70-P, whereas PBS was used as a blank control. Mice were boosted s.c. with above proteins twice at 2-week intervals after the first priming. Two weeks after last immunization, splenocytes were harvested and diluted to different concentrations.

### Enrichment of CD8 ^+^ T cells and natural killer cells from murine splenocytes by MACS-microbead selection

To isolate CD8 ^+^ T cells and natural killer cells from splenocytes harvested from above immunized mice, CD8 ^+^ T cells and natural killer cells microbead kit (Miltenyi Biotec, Bergisch Gladbach, Germany) was used. CD8 ^+^ T cells or natural killer cells from splenocytes captured on antibody-coated magnetic beads were recovered from the magnetic column by eluting with Tris Buffered Saline (TBS) as described by the manufacturer (MS column, Miltenyi Biotec, Bergisch Gladbach, Germany). CD8 ^+^ T cells or natural killer cells-coated beads were pelleted by centrifugation at 1,000g for 10 min. The captured cells were cultured in RPMI 1640 (Gibco-BRL, USA) with 10% fetal bovine serum, 100 U/ml penicillin, and 100 μg/ml streptomycin (Invitrogen Corp., CA, USA) at 37°C under humidified atmosphere of 95% air and 5% CO_2_.

### ELISPOT assay

ELISPOT was used to measure the frequency of cells producing the cytokine IFN-γ in CD8 ^+^ T cells captured from above immunized mice. BD ELISPOT Plates (BD PharMingen, San Diego, CA, USA) were coated with 5 μg/ml rat anti-mouse IFN-γ antibody in 100 μl of PBS. After overnight incubation at 4°C, the wells were washed and blocked with RPMI-1640 culture medium containing 10% fetal bovine serum. 1×10^6^ CD8 ^+^ T cells were added to the ELISPOT plate wells along with 5 μg/ml of AFP peptide containing 10% fetal bovine serum, 10 units/ml of mouse interleukin (IL)-2 (PEPRO Tech ET Ltd.). After culturing at 37°C for 24 h, the plate was washed and then incubated with 2.5 μg/ml biotinylated IFN-γ antibody in 100 μl PBS containing 10% FCS at 4°C overnight. After washing, avidin-HRP in 100 μl PBS was added and incubated for 1 h at room temperature. After washing five times, spots were developed by adding 100 μl 5-bromo-4-chloro-3-indolyl phosphatase/Nitro Blue Tetrazolium (Boehringer Mannheim, Indianapolis, IN, USA). The color spots, representing cytokine producing cells, were counted using an ELISPOT Reader System.

### ELlSA assay

To determine the level of anti-AFP antibody and anti-HSP70 antibody in mice, we examined the serum of mice tail vein after the last immunization by ELISA. A 96-well microplate was coated with 100 μl of 5 μg/ml AFP peptide or HSP70 peptide and incubated at 4°C overnight. The wells were then blocked with PBS containing 5% BSA. Sera were prepared from the mice on day 14 post-immunization, serially diluted in PBS, added to the ELISA wells and incubated at room temperature for 2 h. After washing with PBS-T containing 0.05% Tween-20, the plate was incubated with 1:3000 dilution of a HRP-conjugated goat anti-mouse IgG antibody (Sigma-Aldrich Corp., St Louis, MO, USA) at room temperature for 1 h. The plate was washed five times, developed with O-phenylenediamine away from light at 37°C for 15 min and stopped with 50 μl of 2 M H_2_SO_4_. The ELISA plate was read with a standard ELISA reader at 490 nm. The quantity of antibody was measured in comparison with standard sample diluents.

### Granzyme B and Perforin ELISA

1×10^6^ natural killer cells harvested from above immunized mice were added to the 96 well microplate along with 5 μg/ml of AFP peptide containing 10% fetal bovine serum and 10 units/ml of mouse interleukin (IL)-2 (PEPRO Tech ET Ltd.). After culturing at 37°C for 24 h, granzyme B and perforin released by NK cells was measured using a mouse granzyme B and perforin ELISA kits (Sigma-Aldrich Corp., St Louis, MO, USA) according to manufacturer's instructions. Plates were counted on an ELISA reader at 490 nm.

### Cytotoxic T-lymphocyte (CTL) assays

BALB/C mice were immunized subcutaneously (s.c.) as described above. Two weeks after the last boost, 2.5 × 10^7^ splenocytes were co-cultured with 5 μg/ml of AFP peptide containing 10% fetal bovine serum, 10 units/ml of mouse interleukin (IL)-2 in RPMI 1640 supplemented with 10% FCS at 37°C in 5% CO_2_. After 5 days of stimulation, the viable splenocytes were recovered and used as effector cells, and the H22, Hepa1-6 or MFC, LLC tumor cells were used as target cells. The Non-Radioactive Cytotoxicity Lactate Dehydrogenase (LDH) release assay kit (Promega, USA) was used to measure the cytotoxicity of effector cells against H22, Hepa1-6 or MFC, LLC tumor cells in the ratios of 10:1, 20:1 and 40:1, according to the manufacturer's protocol. Specific lysis was calculated according to the formula: percent specific lysis = ((experimental release value − effector spontaneous release value − target spontaneous release value)/(target maximum release value − target spontaneous release value)) × 100. Results shown are representative of experiments repeated three times.

### CD8 ^+^ T cell depletion CTL assays

To confirm CD8 ^+^ T cell response, a separate set of immunized mice were sacrificed and spleens were collected. CD8 ^+^ T cells were depleted from spleen suspensions with anti-CD8 ^+^ Micro-Beads. The remaining splenocytes were co-cultured with 5 μg/ml of AFP peptide containing 10% fetal bovine serum, 10 units/ml of mouse interleukin (IL)-2 in RPMI 1640 supplemented with 10% FCS at 37°C in 5% CO2. After 5 days of stimulation, the splenocytes were used as effector cells and co-cultured with the H22 or Hepa1-6 tumor cells as described above. Specific lysis was calculated according to the formula: percent specific lysis = ((experimental release value−effector spontaneous release value−target spontaneous release value)/(target maximum release value−target spontaneous release value)) × 100. Results shown are representative of experiments repeated three times.

### Natural killer cell depletion CTL assays

To determine natural killer cell response, another separate set of immunized mice were sacrificed and spleens were collected. Natural killer cells were depleted from spleen suspensions with anti-NKG2D Micro-Beads. The remaining splenocytes were cultured with 5 μg/ml of AFP peptide containing 10% fetal bovine serum, 10 units/ml of mouse interleukin (IL)-2 in RPMI 1640 supplemented with 10% FCS at 37°C in 5% CO2. After 5 days of stimulation, the splenocytes were used as effector cells and co-cultured with the H22 or Hepa1-6 tumor cells as described above. Specific lysis was calculated according to the formula: percent specific lysis = ((experimental release value−effector spontaneous release value−target spontaneous release value)/(target maximum release value−target spontaneous release value)) × 100. Results shown are representative of experiments repeated three times.

### CD8 ^+^ T cell and natural killer cell depletion CTL assays

To further verify whether CD8 ^+^ T cell and natural killer cell concurrently participate in the anti-tumor immunity, a separate set of immunized mice were sacrificed and spleens were collected. CD8 ^+^ T cell and natural killer cells were depleted from spleen suspensions with anti-CD8 ^+^ and anti-NKG2D Micro-Beads. The remaining splenocytes were cultured with 5 μg/ml of AFP peptide containing 10% fetal bovine serum, 10 units/ml of mouse interleukin (IL)-2 in RPMI 1640 supplemented with 10% FCS at 37°C in 5% CO2. After 5 days of stimulation, the splenocytes were used as effector cells and co-cultured with the H22 or Hepa1-6 tumor cells as described above. Specific lysis was calculated according to the formula: percent specific lysis = ((experimental release value−effector spontaneous release value−target spontaneous release value)/(target maximum release value−target spontaneous release value)) × 100. Results shown are representative of experiments repeated three times.

### *In vivo* tumor preventive experiments

To test the ability of HSP70-P/AFP-P conjugated vaccine to inhibit the growth of established tumors, BALB/C mice in each group were injected subcutaneously in the left flank with 5 × 10^5^ H22 or Hepa1-6 tumor cells per mouse on day 0, and then injected subcutaneously in the right flank with different vaccine on day 3, 10, and 17 as described above. H22 or Hepa1-6 tumor cells were washed after enzymatic digestion and resuspended in 0.2 ml of PBS per animal, then injected s.c. into the left flank, while PBS was used as control. The tumor growth was monitored every day. Tumor size was measured in two dimensions with calipers every 3 days one week after tumor inoculation. At each time point, tumor size was determined by measuring the tumor width (a) and length (b). Tumor volume was calculated using the formula: V = (a^2^b)/2. Tumor size measurements were performed by the mean value of each group and performed in duplicate to confirm the results. Percentage of tumor-free mice was recorded and the survival of mice was monitored for eight weeks from the day of tumor challenge.

### Statistical analysis

All statistical analyses were carried out using the SPSS 13.0 statistical software package. The frequencies of IFN-γ-producing splenic cells were valued using *χ*^2^ test. The Student's t test was performed to analyze the significance of differences between final tumor volumes of different groups of animals. *P* < 0.05 was considered statistically significant.
